# Programmed death-1 levels correlate with increased mortality, nosocomial infection and immune dysfunctions in septic shock patients

**DOI:** 10.1186/cc10112

**Published:** 2011-03-21

**Authors:** Caroline Guignant, Alain Lepape, Xin Huang, Hakim Kherouf, Laure Denis, Françoise Poitevin, Christophe Malcus, Aurélie Chéron, Bernard Allaouchiche, François Gueyffier, Alfred Ayala, Guillaume Monneret, Fabienne Venet

**Affiliations:** 1 Hospices Civils de Lyon, Hôpital E. Herriot, Laboratoire d'Immunologie, 5 Place d'Arsonval, 69003 Lyon, France; 2 Hospices Civils de Lyon, CH Lyon-Sud, Service de Réanimation, Chemin du Grand Revoyet, 69495 Pierre-Bénite, France; 3 Division of Surgical Research, Department of Surgery, Brown University School of Medicine/Rhode Island Hospital, 593 Eddy Street, Providence, RI 02903, USA; 4 Hospices Civils de Lyon, CH Lyon-Sud, Laboratoire d'Immunologie, Chemin du Grand Revoyet, 69495 Pierre-Bénite, France; 5 Hospices Civils de Lyon, Hôpital E. Herriot, Service de Réanimation, 5 Place d'Arsonval, 69003 Lyon, France; 6 Hospices Civils de Lyon/INSERM, Centre d'Investigation Clinique (CIC 0201), 52, Boulevard Pinel, 69003 Lyon, France

## Abstract

**Introduction:**

Septic shock remains a major health care problem worldwide. Sepsis-induced immune alterations are thought to play a major role in patients' mortality and susceptibility to nosocomial infections. Programmed death-1 (PD-1) receptor system constitutes a newly described immunoregulatory pathway that negatively controls immune responses. It has recently been shown that PD-1 knock-out mice exhibited a lower mortality in response to experimental sepsis. The objective of the present study was to investigate PD-1-related molecule expressions in septic shock patients.

**Methods:**

This prospective and observational study included 64 septic shock patients, 13 trauma patients and 49 healthy individuals. PD-1-related-molecule expressions were measured by flow cytometry on circulating leukocytes. Plasmatic interleukin (IL)-10 concentration as well as *ex vivo *mitogen-induced lymphocyte proliferation were assessed.

**Results:**

We observed that septic shock patients displayed increased PD-1, PD-Ligand1 (PD-L1) and PD-L2 monocyte expressions and enhanced PD-1 and PD-L1 CD4^+ ^T lymphocyte expressions at day 1-2 and 3-5 after the onset of shock in comparison with patients with trauma and healthy volunteers. Importantly, increased expressions were associated with increased occurrence of secondary nosocomial infections and mortality after septic shock as well as with decreased mitogen-induced lymphocyte proliferation and increased circulating IL-10 concentration.

**Conclusions:**

These findings indicate that PD-1-related molecules may constitute a novel immunoregulatory system involved in sepsis-induced immune alterations. Results should be confirmed in a larger cohort of patients. This may offer innovative therapeutic perspectives on the treatment of this hitherto deadly disease.

## Introduction

Sepsis remains a major health-care problem worldwide [1]. For example, during the last decade, its hospitalization rate has almost doubled in the US [2]. This is associated with a mortality rate approaching 50% in the case of septic shock [3,4], despite the development of novel treatments such as early appropriate antibiotherapy, early goal-directed therapy, and activated protein C. Therefore, a better understanding of pathophysiology of severe sepsis is a necessity if we are to decrease the high mortality rate of this condition.

Septic pathophysiology is a culmination of multiple complex dynamic processes whose interactions are only partially understood. However, it is now accepted that after a rapid proinflammatory response, a counter-regulatory phase characterized by immune alterations impacting both innate and adaptive responses develops [1,5,6]. This second phase has been characterized by an increased production of anti-inflammatory cytokines (mainly interleukin-10 (IL-10) and transforming growth factor-beta) [7], increased lymphocyte apoptosis [8], increased proportion of circulating regulatory T cells [9], and a severe downregulation of monocyte HLA-DR expression [10]. However, much remains to be understood in order to clarify our vision of this complex and multiparameter pathophysiologic process.

Programmed death-1 (PD-1)-related molecules constitute a complex system of negative regulators involved in controlling T-cell responses. This system is composed of PD-1 (CD279) and its two ligands, PD-L1 (B7-H1, CD274) and PD-L2 (B7-DC, CD273). These molecules belong to the B7:CD28 family [11]. They are best understood relative to their role in viral infections and oncology [11-14]. It has been proposed that pathogens and tumor cells may take advantage of this pathway to escape the host's immune defenses. Considering their immunoregulatory properties, we postulated that the PD-1 system could participate in sepsis-induced immune dysfunctions. Indeed, it was recently shown that PD-1 knockout mice exhibited not only a greater capacity to clear bacteria but, more importantly, a lower mortality in response to experimental sepsis [15]. Therefore, the objective of this study was to investigate the PD-1 system in patients with septic shock.

## Materials and methods

### Patients

After Hospices Civils de Lyon (Lyon, France) ethics committee review and approval, we enrolled 64 patients with septic shock in this observational clinical study (from 2007 to 2009). Septic shock was diagnosed according to the diagnostic criteria of the American College of Chest Physicians/Society of Critical Care Medicine [16]. Patients were admitted to one of the two intensive care units (ICUs) (one medical, the other surgical) of the Lyon-Sud University Hospital (France).

Septic shock was defined by an identifiable site of infection, which was evidence of a systemic inflammatory response manifested by at least two of the following criteria: (a) temperature of greater than 38°C or less than 36°C, (b) heart rate of greater than 90 beats per minute, (c) respiratory rate of greater than 20 breaths per minute, and (d) white blood cell count of greater than 12,000 or less than 4,000/mm^3 ^and hypotension persisting despite fluid resuscitation and requiring vasopressor therapy. The beginning of vasopressive therapy was considered the time of diagnosis of septic shock. Exclusion criteria were age of less than 18 years and the absence of circulating leukocytes for flow cytometry phenotyping. No patients with HIV were included. Patients with cancer were excluded from our study if they presented with an aplasia (defined by a polymorphonuclear neutrophil count of less than 0.5 G/L) or were treated with a high dose of corticoids (estimated as treatment superior to 10 mg equivalent prednisolone/day or more than 700 mg equivalent prednisolone accrued the first day of inclusion) or both.

The following clinical and biological data were collected: demographic characteristics (age and gender), admission category (elective or emergency surgery and medicine), referral pattern (community-, hospital-, or ICU-acquired septic shock), microbiological findings, clinical scores (Simplified Acute Physiology Score II (SAPS II) and sepsis-related organ failure assessment (SOFA) score), incidence of secondary nosocomial infections (defined as microbiologically documented pulmonary infection, urinary tract infection, bloodstream infection, and catheter-related infection that occurred 48 hours after ICU admission and up to ICU discharge [17]), and the outcome after 28 days (death or survival).

The protocol was reviewed by the institutional ethics committee, which waived the need for informed consent because the study was observational and involved sampling of very small quantities of blood. The purpose of the study was explained to the patients or members of their families. Samples were collected from residual blood after completion of routine follow-up. Ethylenediaminetetraacetic acid (EDTA)-anti-coagulated blood was collected from patients at different time points: day (D) 1-2, D3-5, and D6-10 after diagnosis of septic shock. Additionally, 13 trauma patients were included in the study within the first 48 hours of admission. Inclusion criteria were trauma, age of at least 18 years, and an initial injury severity score (ISS) of at least 25. Finally, 49 healthy volunteers from laboratory staff of our hospital were included as controls.

### Flow cytometry reagents

The following antibodies were used: PC5-labeled anti-CD4, PC5-labeled anti-CD8, PC5-labeled anti-CD14, PC5-labeled anti-CD25, PE-labeled anti-CD127, FITC-labeled anti-CD14, ECD-labeled anti-CD4 (Beckman Coulter, Miami, FL, USA), and PE-labeled anti-HLA-DR or its isotype PE-labeled IgG2a (Becton-Dickinson Biosciences, San Jose, CA, USA), PE-labeled anti-human CD249 (PD-1, clone MIH4), FITC-labeled anti-human CD274 (PD-L1, clone MIH1), or PE-labeled anti-human CD273 (PD-L2, clone MIH18) (BD Biosciences). Red blood cells were lysed using the automated TQ-Prep (Beckman Coulter) or using FACS-lysing solution (BD Biosciences). Samples were run on FC500 (Beckman Coulter) and analyzed using CXP software (Beckman Coulter).

### Plasma cytokine measurements

IL-10 concentration in patients' plasma samples was measured by Bio-Plex Pro Assays (Bio-Rad Laboratories, Inc., Hercules, CA, USA). Unknown sample values presented as picograms per milliliter were determined against human standards as described by the manufacturer.

### Cell isolation, culture conditions, and cell proliferation assay

In brief, peripheral blood mononuclear cells (PBMCs) were isolated by Ficoll density gradient centrifugation (PAA Laboratories, Pasching, Austria). PBMCs were washed three times in phosphate-buffered saline (bioMérieux, Marcy-l'Etoile, France) and resuspended in complete medium - that is, RPMI supplemented with HEPES (25 mM), sodium bicarbonate (2 g/L) (Eurobio Laboratories, Les Ulis, France), 10% human serum AB (obtained from a pool of healthy volunteers), 2 mM L-glutamine (Lonza, Verviers, Belgium), 20 UI/mL penicillin, 20 μg/mL streptomycin (Sigma-Aldrich, St. Louis, MO, USA), and 2.5 μg/mL Amphotericin B (Bristol-Myers Squibb Company, Princeton, NJ, USA). Cells were kept on ice until stainings or cell cultures were performed.

PBMCs were seeded at a density of 1 × 10^6 ^cells/mL (50,000 cells/well, 100 μL) in flat-bottom 96-well microtiter plates and were stimulated with 5 μg/mL phytohemagglutinin (PHA) (Remel, part of Thermo Fisher Scientific, Lenexa, KS, USA). Cells were incubated 48 hours at 37°C in a humidified 5% CO_2 _atmosphere.

[methyl-^3^H]-Thymidine (20 μCi/mL) (PerkinElmer, Waltham, MA, USA) was added 24 hours before harvesting cells on fiberglass filters by means of an automated cell harvester (PerkinElmer). Incorporated radioactivity was measured in a direct beta counter (PerkinElmer). Assays were carried out in triplicate.

### Data analysis and statistics

Patients' clinical and biological parameters were presented as frequencies, percentages, medians, and interquartile ranges (IQRs). Differences in expression levels were calculated using the Mann-Whitney *U *test or, when multiple comparisons were performed, the Friedman test. Correlations were calculated using the Spearman rank test. *P *values of not more than 0.05 were considered statistically significant; if necessary, correction for the number of tests was performed. Statistical analysis was performed using SPSS software (version 12.0; SPSS Inc., Chicago, IL, USA).

## Results

### Clinical characteristics of the patient population

Sixty-four patients with septic shock (20 women and 44 men) were included in the study. Their clinical characteristics are shown in Table 1. Median age at admission was 63 years (IQR 54 to 73). Median values for SAPS II and SOFA score at diagnosis of shock were 53 (IQR 39 to 64) and 10 (IQR 8 to 12), respectively, indicating a high level of severity. Approximately 30% of patients developed secondary nosocomial infections, and 28-day mortality was 17%.

**Table 1:** Clinical characteristics of the patients with septic shock

Parameters	Patients with septic shock (*n *= 64)
Age at admission, years	63 (54-73)
Males, number (percentage)	44 (68.8)
SAPS II at diagnosis of shock	53 (39-64)
Main admission category, number (percentage)	
Medical	25 (39.1)
Surgery + trauma	39 (60.9)
Comorbidities, number (percentage) of patients	
None	35 (54.7)
One or more	29 (45.3)
SOFA score at diagnosis of shock	10 (8-12)
28-day non-survivors, number (percentage)	11 (17.2)
Infection, number (percentage)	
Diagnosis	
Radiology	10 (15.6)
Surgery	7 (10.9)
Microbiologically documented	
Bacilli Gram-negative	26 (40.6)
Cocci Gram-positive	30 (46.9)
Fungi	8 (12.5)
Type of infection	
Community-acquired	38 (59.4)
Nosocomial	26 (40.6)
Site of infection	
Pulmonary	21 (32.8)
Abdominal	27 (42.2)
Others	16 (25)
Secondary nosocomial infections, number (percentage)	19 (29.7)
Immunological parameters	
Percentage mHLA-DR^a^	45.5 (29.5-69.5)
CD4^+ ^T-cell counts, cells/μL^a^	319 (226-681)
Percentage of regulatory T cells^a^	8.5 (6.1-11.2)

Values are presented as median and interquartile range (IQR) for continuous variables or as number of cases and percentage for categorical data. ^a^Measured at day 3 to 5 after the onset of septic shock. CD4^+ ^T-cell counts were measured in 41 patients with septic shock, and percentage of regulatory T cells (CD4^+^CD25^+^CD127^-^) was measured in 42 patients. mHLA-DR, monocyte HLA-DR; SAPS II, Simplified Acute Physiology Score II; SOFA, sepsis-related organ failure assessment.

Septic patients presented with typical features of sepsis-induced immunosuppression and displayed a reduced monocyte HLA-DR expression at D3-5 (median value 45.5%, IQR 29.5 to 69.5) in comparison with control values (>90% [18]). Median CD4^+ ^T-cell count was also decreased in patients in comparison with healthy volunteers (319 cells/μL (IQR 226 to 681) versus 822 cells/μL (IQR 679 to 1,075), respectively; *P *< 0.001), whereas percentage of circulating regulatory T cells (CD4^+^CD25^+^CD127^- ^T lymphocytes) was augmented (8.5% (IQR 6.1% to 11.2%) versus 6.2% (IQR 5.2% to 7.6%), respectively; *P *= 0.001).

Thirteen trauma patients (9 men and 4 women) were also included in the study. Median age at admission was 34 years (IQR 24 to 56). In the first 24 hours of admission, they presented a median ISS of 32 (IQR 26 to 34) and a median SAPS II of 39 (IQR 22 to 52).

### PD-1-related molecule expression in patients with septic shock

**Figure 1:**
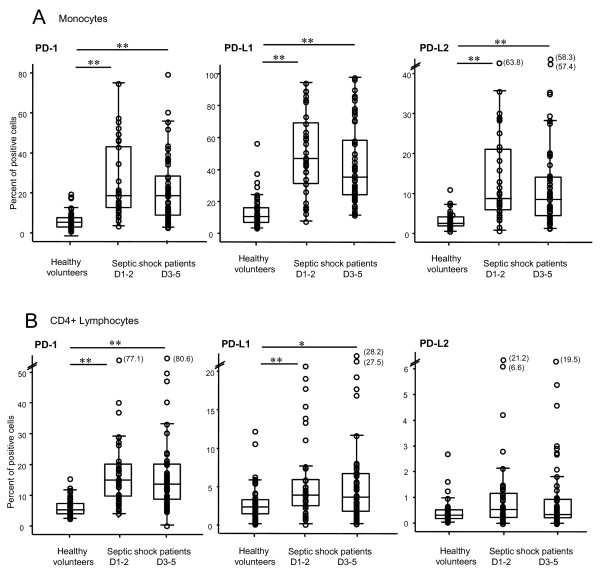
**PD-1, PD-L1, and PD-L2 measurements on circulating CD4^+ ^lymphocytes and monocytes in septic shock patients and healthy volunteers**. PD-1-related molecule expressions were measured on circulating monocytes **(a) **and CD4^+ ^lymphocytes **(b) **in whole blood from healthy volunteers (*n *= 49) and septic shock patients at day 1 to 2 (D1-2) (*n *= 37) and at day 3 to 5 (D3-5) (*n *= 56) after the onset of shock. Results are presented as percentages of positive cells among total population of monocytes or CD4^+ ^lymphocytes and as box-plots and individual values. *P < 0.020, **P ≤ 0.002 (Mann-Whitney *U *test). A *P *value of less than 0.025 was considered statistically significant (with correction for the number of tests). PD-1, programmed death-1; PD-L1, programmed death-ligand 1; PD-L2, programmed death-ligand 2.

PD-1, PD-L1, and PD-L2 expressions were measured on circulating CD4^+ ^lymphocytes, CD8^+ ^lymphocytes (PD-1 only), and monocytes at D1-2 and 3-5 after the onset of septic shock. Results for CD4^+ ^lymphocytes and monocytes are shown in Figure 1.

The percentages of circulating monocytes expressing PD-1, PD-L1, or PD-L2 were markedly increased in patients with septic shock in comparison with healthy volunteers during the overall monitoring (Figure 1a). This augmentation was present for PD-1 (median control values: 5.0% versus 18.6% (D1-2) and 17.8% (D3-5) in patients; *P *< 0.001), for PD-L1 (control values: 10.2% versus 46.6% (D1-2) and 34.9% (D3-5) in patients; *P *< 0.001), and for PD-L2 (control values: 2.6% versus 8.7% (D1-2) and 8.5% (D3-5) in patients; *P *< 0.001). Similar results were observed when flow cytometry data were expressed as mean fluorescence intensity (MFI) (Table 2). In trauma patients, PD-1-related molecule expressions on monocytes were significantly increased in comparison with healthy individuals (for PD-1: control value: 5.0% versus 9.6%, *P *= 0.005; for PD-L1: control value: 10.2% versus 40.1%, *P *< 0.001; and for PD-L2: control value: 2.6% versus 7.2%, *P *< 0.001). However, PD-1 expression on monocytes was significantly lower in trauma than in septic shock patients at D1-2 (9.6% versus 18.6%, respectively; *P *= 0.008) (data not shown).

**Table 2:** PD-1-related molecule expressions as mean of fluorescence intensity on leukocytes in septic shock patients and healthy volunteers

			CD4^+ ^T cells			CD8^+ ^T cells	Monocytes		
					
			PD-1	PD-L1	PD-L2	PD-1	PD-1	PD-L1	PD-L2
Healthy volunteers		Median	8.7	11.5	4.9	13.6	12.3	16.9	8.9
		IQR	(7.8-10.5)	(10.1-12.0)	(4.5-5.6)	(11.1-20.4)	(10.1-15.8)	(15.3-18.2)	(7.7-9.8)
		Median	13.1	11.4	6.0	18.1	17.4	22.0	11.6
	Day 1-2	IQR	(11.4-19.7)	(9.8-14.3)	(4.8-7.1)	(13.6-24.4)	(14.6-24.0)	(19.3-31.8)	(9.9-13.6)
Septic shock patients		*P *value	<0.001	0.150	0.009	0.213	<0.001	<0.001	<0.001
		Median	12.2	11.4	5.4	17.5	16.2	21.1	11.1
	Day 3-5	IQR	(10.8-15.7)	(10.0-13.5)	(4.4-7.1)	(11.8-22.3)	(13.0-20.4)	(18.2-28.0)	(9.6-13.3)
		*P *value	<0.001	0.289	0.232	0.306	<0.001	<0.001	<0.001

Programmed death-1 (PD-1)-related molecule expressions were measured on circulating CD4^+ ^and CD8^+ ^lymphocytes and monocytes in whole blood from healthy volunteers (*n *= 49) and septic shock patients at day 1 to 2 (*n *= 37) and at day 3 to 5 (*n *= 56) after the onset of shock. Results are presented as mean fluorescence intensity. A *P *value of less than 0.025 was considered statistically significant, and correction for the number of tests was performed (Mann-Whitney *U *test). IQR, interquartile range.

Likewise, the percentages of circulating CD4^+ ^lymphocytes expressing PD-1 or PD-L1 were notably increased in patients with septic shock in comparison with healthy volunteers during the overall monitoring (for PD-1: control values: 5.4% versus 15.0% (D1-2) and 13.6% (D3-5), *P *< 0.001; for PD-L1: control values: 2.5% versus 3.9% (D1-2; *P *= 0.002) and 3.6% (D3-5; *P *= 0.016) in patients) (Figure 1b). Alternatively, no significant differences were observed between patients and healthy volunteers for percentages of CD4^+ ^cells expressing PD-L2 (Figure 1b) or of CD8^+ ^lymphocytes positive for PD-1 (Table 2). Once again, similar results were observed when flow cytometry results were expressed as MFI (Table 2). No difference in PD-1-related molecule expressions was observed between trauma patients and healthy individuals. However, the percentage of PD-1 expressing CD4^+ ^cells was significantly lower in trauma than in septic shock patients at D1-2 (5.2% versus 15.0%, respectively; *P *< 0.001) (data not shown).

Of note, there was no variation of PD-1-related molecule expressions in regard to age or gender either in healthy subjects or in patients with septic shock. Indeed, we did not observe significant correlations between PD-1-related molecule expressions and the age of septic shock patients (*r *= 0.21, *P *= 0.12 for PD-1 expression on CD4^+ ^lymphocytes; *r *= 0.04, *P *= 0.78 for PD-L1 expression on monocytes) or of healthy volunteers (*r *= 0.10, *P *= 0.49 for PD-1 expression on CD4^+ ^lymphocytes; *r *= -0.15, *P *= 0.30 for PD-L1 expression on monocytes).

**Figure 2:**
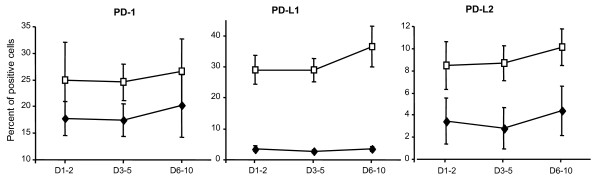
**Sequential PD-1, PD-L1, and PD-L2 measurements on circulating CD4^+ ^lymphocytes and monocytes in patients with septic shock**. In 10 patients with septic shock, sequential blood samples were obtained at day 1 to 2 (D1-2), day 3 to 5 (D3-5), and day 6 to 10 (D6-10) after the onset of shock, and percentages of PD-1-, PD-L1-, and PD-L2-positive CD4^+ ^lymphocytes (black diamonds) and monocytes (white squares) were measured by flow cytometry. Results are expressed as mean ± standard error of the mean. The Friedman test was performed: *P *values were greater than 0.05 for all of the analyses. PD-1, programmed death-1; PD-L1, programmed death-ligand 1; PD-L2, programmed death-ligand 2.

Finally, in 10 patients with septic shock, sequential blood samples were obtained at D1-2, D3-5, and D6-10 after the onset of shock. During this period, no significant variations over time in regard to PD-1 molecule expressions either on monocytes or on lymphocytes were observed (Figure 2).

### Association between PD-1-related molecule expressions and clinical parameters

**Figure 3:**
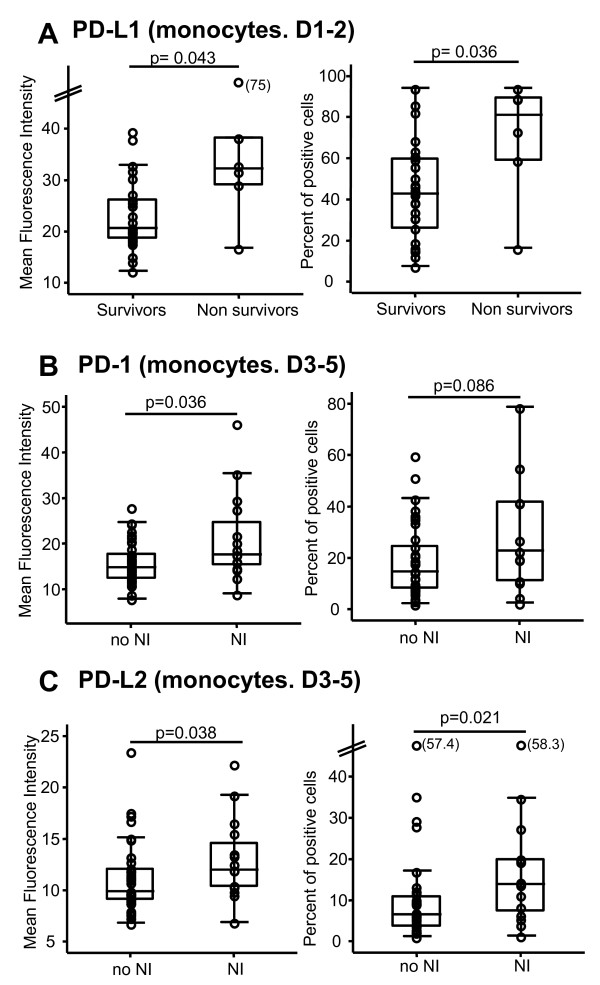
**PD-1-related molecule expressions on monocytes and clinical outcomes**. **(a) **Monocyte PD-L1 expression was measured on 26 survivors and 6 non-survivors at day 1 to 2 (D1-2) after the onset of septic shock. Monocyte PD-1 **(b) **and PD-L2 **(c) **expressions were measured at day 3 to 5 (D3-5) after the onset of shock on 15 patients who developed a secondary nosocomial infection during their intensive care unit stay (NI) and 38 patients who remained free of secondary infection (no NI). Flow cytometry data are expressed as (left) mean fluorescence intensities and (right) percentages of positive cells out of total circulating monocytes. Results are presented as box-plots as well as individual values. The Mann-Whitney *U *test was performed. PD-1, programmed death-1; PD-L1, programmed death-ligand 1; PD-L2, programmed death-ligand 2.

To assess the clinical relevance of the increase in PD-1-related molecule expressions after septic shock, flow cytometric measurements were correlated with clinical parameters and usual biomarkers of sepsis-induced immunosuppression. No significant correlations were found between PD-1-related molecule expressions and percentages of HLA-DR expressing monocytes, CD4^+ ^lymphocyte count, percentage of circulating regulatory T cells, or severity scores calculated at the onset of shock (SAPS II or SOFA score) (data not shown). However, at D1-2, we observed that PD-L1 expression on monocytes was significantly higher in non-survivors in comparison with survivors (Figure 3a). Moreover, at D3-5, patients who went on to develop a secondary nosocomial infection presented with higher PD-1 (Figure 3b) and PD-L2 (Figure 3c) expressions on their blood monocytes in comparison with those who remained free of any secondary nosocomial episode.

### Correlation between plasma IL-10 concentration and PD-1-related molecule expression in patients with septic shock

**Figure 4:**
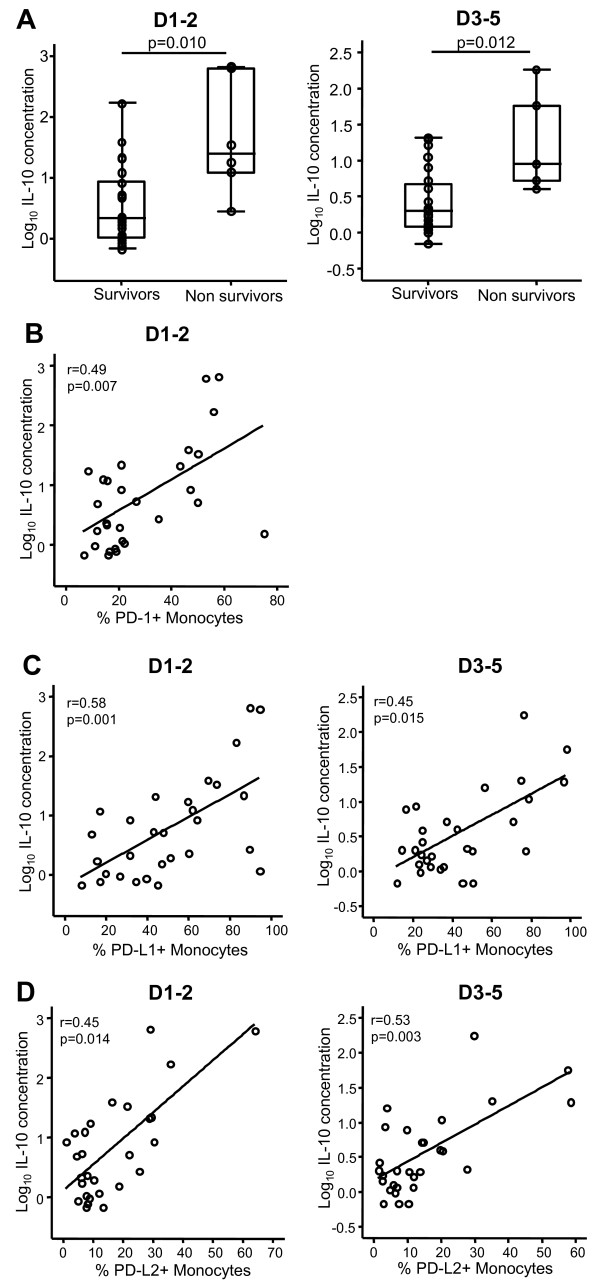
**Plasma IL-10 concentration and PD-1 expression in patients with septic shock**. **(a) **Plasma IL-10 concentration was measured in survivors and non-survivors at day 1 to 2 (D1-2) (*n *= 23 and *n *= 6, respectively) and at day 3 to 5 (D3-5) (*n *= 24 and *n *= 5, respectively) after septic shock. Results are presented as box-plots and as individual values, and horizontal lines represent medians. The Mann-Whitney *U *test was performed. **(b-d) **Correlations between increased plasma IL-10 concentration and increased PD-1 (b), PD-L1 (c), and PD-L2 (d) expressions on monocytes were calculated at D1-2 and D3-5 in 29 patients with septic shock. The Spearman correlation test was used to assess statistical significance. IL-10, interleukin-10; PD-1, programmed death-1; PD-L1, programmed death-ligand 1; PD-L2, programmed death-ligand 2.

Increased circulating IL-10 concentration has been linked with mortality after septic shock [19] and recently with enhanced PD-1 expression in HIV-infected patients [20]. We thus measured circulating IL-10 levels in 29 septic shock patients for whom plasma samples were available and we correlated this parameter with leukocyte PD-1/PD-L expressions. Not surprisingly, we observed that non-survivors exhibited higher plasma IL-10 concentration than survivors at D1-2 and D3-5 (*P *= 0.01 for both) (Figure 4a). Interestingly, a significant positive correlation was measured between PD-1 monocyte expression and plasma IL-10 concentration in patients at D1-2 (*r *= 0.49; *P *= 0.007) (Figure 4b) but not at D3-5 (data not shown). In addition, significant correlations were observed between both PD-L1 or PD-L2 monocyte expressions and increased plasma IL-10 concentration at D1-2 (*r *= 0.58; *P *= 0.001 and *r *= 0.45; *P *= 0.014, respectively) and D3-5 (*r *= 0.45; *P *= 0.015 and *r *= 0.53; *P *= 0.003, respectively) (Figure 4c, d). Of note, no correlations were found between PD-1/PD-L-related molecule expressions on CD4^+ ^lymphocytes and changes in plasma IL-10 concentration (data not shown). Also, for all of these observations made for percentage of positive cells, similar correlations were obtained when flow cytometry results were expressed as MFI (data not shown).

### Decreased lymphocyte proliferation after septic shock

**Figure 5:**
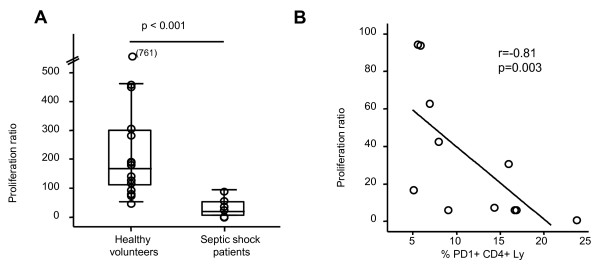
**Lymphocyte proliferation and PD-1 expression in septic shock patients and healthy volunteers**. **(a) **Lymphocyte proliferation was measured in 16 healthy volunteers and 11 septic shock patients (at day 3 to 5, or D3-5) by ^3^H-thymidine incorporation after stimulation with phytohemagglutinin (5 μg/mL). The proliferation ratio was calculated as the ratio between the numbers of count per minute in the stimulated wells, divided by non-stimulated wells. Results are presented as box-plots as well as individual values. Statistical significance was calculated using the Mann-Whitney *U *test. **(b) **The correlation between percentages of PD-1^+^CD4^+ ^lymphocytes (Ly) and proliferation ratio was assessed in 11 patients with septic shock at D3-5. The Spearman correlation test was performed. PD-1, programmed death-1.

In an attempt to begin to address the biological significance of these changes in PD-1 expression to the development of sepsis-induced lymphocyte dysfunction, freshly isolated PBMCs from septic shock patients and healthy volunteers were assessed for their capacity to respond to PHA. As expected, we observed that lymphocyte proliferation was significantly reduced in patients in comparison with healthy volunteers (*P *< 0.001) (Figure 5a). Interestingly, in patients, a significant negative correlation was observed between this reduced proliferation and PD-1 (*r *= -0.81 with *P *= 0.003) (Figure 5b) or PD-L1 (*r *= -0.63 with *P *= 0.039) (data not shown) overexpression on circulating CD4^+ ^lymphocytes. Similar results were obtained when PD-1 and PD-L1 staining was expressed as MFI (*r *= -0.80 with *P *= 0.003 and *r *= -0.63 with *P *= 0.038, respectively).

## Discussion

PD-1 and its ligands, PD-L1 and PD-L2, belong to the B7-CD28 family of molecules [11]. Co-ligation of T-cell receptor with the PD-1 system is thought to induce an inhibitory signal in T cells characterized by cell cycle arrest, inability to proliferate, and reduced cytokine synthesis (interferon-gamma (IFN-γ) or IL-2 or both [21-24]). The co-inhibitory PD-1 system has been studied mainly in viral diseases and oncology. This system may be used by viral pathogens or cancer cells to evade the host's immune response [11]. Of note, in virus-infected patients, CD8^+ ^T cells overexpressing PD-1 (in comparison with healthy volunteers) exhibit a so-called 'exhaustion profile' as they produced less IFN-γ following antigen stimulation, had reduced cytotoxic activity, and had decreased proliferation in response to specific antigens [25-27].

Interestingly, we demonstrated here for the first time that typical sepsis-immune dysfunctions such as decreased monocyte HLA-DR expression, decreased circulating CD4^+ ^T-cell count, and increased percentage of regulatory T cells [6] were associated with an increased PD-1 expression on CD4^+ ^lymphocytes (and PD-L1 to a lesser extent) and increased PD-1, PD-L1, and PD-L2 expressions on monocytes. Of note, during the review of this article, a study including 19 patients with septic shock confirmed that PD-1 expression on CD4^+ ^lymphocytes and PD-L1 expression on monocytes were elevated in comparison with healthy volunteers [28]. Moreover, we observed a significant inverse correlation between increased PD-1 and PD-L1 CD4^+ ^lymphocyte expressions and decreased PHA-induced lymphocyte proliferation in patients with septic shock. Such inverse correlations have been described in patients with hepatitis B [29] and in patients with HIV [14]. Additionally, we observed a significant correlation between increased plasma IL-10 concentration and increased PD-1-related molecule expressions on monocytes from patients with septic shock. Recently, in an HIV-infected patient cohort, such a correlation was described and implicated in the reduced CD4^+ ^T-cell proliferation observed in these patients [20]. In accordance with these observations, we recently showed not only that the increased septic blood levels of IL-10 are reduced but also that the rise in lipopolysaccharide-induced IL-10 release by septic mouse macrophages is lost in animals that are genetically deficient (knockout) in functional PD-1 [15]. Overall, our results therefore suggest a link between increased PD-1-related molecule expressions and the development of sepsis-induced immune dysfunctions.

Surprisingly, we found no PD-1 overexpression on circulating CD8^+ ^T cells in septic patients. This is divergent from the observations made in patients with HIV, hepatitis B virus, or hepatitis C virus [13,25,26,29]. One explanation may be that CD8^+ ^cells, which play a prominent role in viral infections, may be less central to the response patients make to septic shock. This is because this response is thought mainly to be a response to a bacterial challenge. Of note, Zhang and colleagues [28] recently described an increased PD-1 expression on CD8^+ ^lymphocytes in a small cohort of 19 septic shock patients in comparison with healthy volunteers. Thus, this observation deserves to be further examined in a larger cohort of septic patients.

Of note, in our cohort, non-survivors displayed higher monocyte PD-L1 expression in comparison with survivors, and patients who went on to develop secondary nosocomial infections had significantly higher PD-1 and PD-L2 monocyte expressions in comparison with patients who remained free of secondary infection. This is consistent with data observed in a murine model of sepsis, in which after the induction of polymicrobial septic shock by cecal ligation and puncture (CLP), PD-1 knockout mice showed a markedly improved capacity to clear bacteria, both at the local (peritoneal lavage) and the systemic (blood) level, in comparison with wild-type mice [15]. Moreover, PD-L1 blockade significantly improved survival, prevented sepsis-induced depletion of lymphocytes, increased tumor necrosis factor-alpha and IL-6 productions, decreased IL-10 production, and enhanced bacterial clearance in mice after CLP [30]. Similar data were recently observed *ex vivo *in patients with septic shock [28]. Importantly, we show here that the PD-1 system not only may play a role in immune dysfunction but also may be an indicator of septic mortality and subsequent infectious episodes in septic patients.

Increased expressions of co-inhibitory as well as decreased expressions of co-stimulatory members of the B7-CD28 family of molecules have been described in ICU patients. In trauma patients, CTLA-4 and PD-1 expressions were elevated in anergic T cells [31]. Similar results were observed at the mRNA level in trauma patients with multiple organ dysfunction syndrome [32]. In mice, it was recently shown that B- and T-lymphocyte attenuator (BTLA) (another co-inhibitory molecule) was induced at the early phase of *Listeria monocytogenes *infection [33]. Moreover, CD3 expression on T lymphocytes was reduced in septic shock patients in comparison with healthy volunteers [34]. Similar decreased expression was observed at the mRNA level in patients developing sepsis or severe sepsis postoperatively [35] and in trauma patients [36]. Finally, CD28 expression (delivering a positive co-signal after ligation to B7.1 or B7.2) was depressed in trauma patients' anergic T cells and may contribute to incomplete activation of these cells [36]. In total, these alterations may play a major role in lymphocyte anergy that has been observed in ICU patients and that has been associated with increased mortality and risk of nosocomial infections. They could thus represent potential therapeutic targets and associated markers to guide future immunotherapeutic decisions [37].

The present study has some limitations. We could not address the involvement of the PD-1 system in sepsis-induced apoptosis. Indeed, PD-1 was first described as being implicated in programmed cell death [38]. It was also recently described that PD-1^+^CD8^+ ^T cells were more sensitive to both spontaneous and Fas-induced apoptosis in comparison with PD-1^-^CD8^+ ^T cells [14]. Most interestingly, it has recently been reported that *in vivo *blockade of PD-1 could decrease T- and B-cell apoptosis and improve survival in CLP-induced septic mice [39]. However, given the technical difficulties encountered in the measurement of apoptosis in clinical samples, let alone in those of minimal-volume septic shock patients' whole blood samples that are already dedicated to numerous assays [40], this aspect could not be specifically addressed here and thus deserves to be investigated in studies specifically dedicated to examining that process/index.

## Conclusions

We describe here for the first time that PD-1/PD-L-related molecule expression is markedly induced on circulating cells of patients with septic shock. Moreover, increased PD-1-related molecule expression appears to be correlated with the development of immune dysfunctions, secondary nosocomial infections, and death. We believe that, although these findings need to be confirmed in a larger multicentered clinical study, our results are in line with the recent commentary of Hotchkiss and Opal [37], which proposes the use of anti-PD-1 blocking antibodies in septic patients given that these molecules are already being tested (and well tolerated) in clinical trials in patients with cancer. Although this hypothesis remains a speculation at the moment and further functional studies are required to understand the mechanism of action of PD-1-related molecules in patients with septic shock, the PD-1 family of receptor and ligands could represent a potential innovative therapeutic strategy with which to restore immune functions and may further alter morbidity/mortality seen with sepsis, and this is in line with the concept of tailored immunotherapy [41]. Through their changing expression (alone or together with other markers), PD-1 molecules could give us insight into the immune status of the septic individual as well as their possible responsiveness to various established or novel therapeutic approaches (or both) used in these critically ill patients.

## Key messages

• Programmed death-1 (PD-1)-related molecule expressions are increased on circulating monocytes and CD4^+ ^lymphocytes after septic shock in comparison with healthy volunteers and trauma patients.

• Increased PD-1-related molecule expressions on monocytes are significantly associated with increased mortality and occurrence of secondary nosocomial infections after septic shock.

• Augmented PD-1-related molecule expressions after septic shock are associated with immune dysfunctions such as decreased mitogen-induced lymphocyte proliferation and increased circulating interleukin-10 concentration.

## Abbreviations

CLP: cecal ligation and puncture; D: day; ICU: intensive care unit; IFN-γ: interferon-gamma; IL: interleukin; IQR: interquartile range; ISS: injury severity score; MFI: mean fluorescence intensity; PBMC: peripheral blood mononuclear cell; PD-1: programmed death-1; PD-L1: programmed death-ligand 1; PD-L2: programmed death-ligand 2; PHA: phytohemagglutinin; SAPS II: Simplified Acute Physiology Score II; SOFA: sepsis-related organ failure assessment.

## Competing interests

The authors declare that they have no competing interests.

## Authors' contributions

CG, FV, GM, and AL designed the study, collected clinical information, analyzed raw data, performed statistical analysis, and contributed to writing the paper. HK, FP, CM, and LD performed the immunological monitoring. AA, FG, and XH designed the study and contributed to writing the paper. AC and BA collected clinical information about trauma patients. All the authors read and approved the final version of the manuscript.
